# AGEs-Induced Calcification and Apoptosis in Human Vascular Smooth Muscle Cells Is Reversed by Inhibition of Autophagy

**DOI:** 10.3389/fphar.2021.692431

**Published:** 2021-10-20

**Authors:** Hu-Qiang He, Yuan-Qing Qu, Betty Yuen Kwan Law, Cong-ling Qiu, Yu Han, Ivo Ricardo de Seabra Rodrigues Dias, Yong Liu, Jie Zhang, An-Guo Wu, Cheng-Wen Wu, Simon Wing Fai Mok, Xin Cheng, Yan-Zheng He, Vincent Kam Wai Wong

**Affiliations:** ^1^ Dr. Neher’s Biophysics Laboratory for Innovative Drug Discovery, State Key Laboratory of Quality Research in Chinese Medicine, Macau University of Science and Technology, Macau, China; ^2^ Department of Vascular Surgery, Affiliated Hospital of Southwest Medical University, Luzhou, China; ^3^ Guangdong-Hong Kong-Macau Joint Lab on Chinese Medicine and Immune Disease Research, Macau, China; ^4^ Department of Nuclear Medicine, Affiliated Hospital of Southwest Medical University, Luzhou, China; ^5^ Nuclear Medicine and Molecular Imaging Key Laboratory of Sichuan Province, Luzhou, China; ^6^ Laboratory of Chinese Materia Medical, School of Pharmacy, Southwest Medical University, Luzhou, China; ^7^ Institute of Cardiovascular Research, The Key Laboratory of Medical Electrophysiology, Collaborative Innovation Center for Prevention and Treatment of Cardiovascular Disease of Sichuan Province, Southwest Medical University, Luzhou, China; ^8^ Affiliated Hospital of Ya’an Polytechnic College, Ya’an, China

**Keywords:** autophagy, ATG7, ages, HASMCs, calcification, apoptosis

## Abstract

Vascular calcification (VC) in macrovascular and peripheral blood vessels is one of the main factors leading to diabetes mellitus (DM) and death. Apart from the induction of vascular calcification, advanced glycation end products (AGEs) have also been reported to modulate autophagy and apoptosis in DM. Autophagy plays a role in maintaining the stabilization of the external and internal microenvironment. This process is vital for regulating arteriosclerosis. However, the internal mechanisms of this pathogenic process are still unclear. Besides, the relationship among autophagy, apoptosis, and calcification in HASMCs upon AGEs exposure has not been reported in detail. In this study, we established a calcification model of SMC through the intervention of AGEs. It was found that the calcification was upregulated in AGEs treated HASMCs when autophagy and apoptosis were activated. In the country, AGEs-activated calcification and apoptosis were suppressed in Atg7 knockout cells or pretreated with wortmannin (WM), an autophagy inhibitor. These results provide new insights to conduct further investigations on the potential clinical applications for autophagy inhibitors in the treatment of diabetes-related vascular calcification.

## Introduction

Diabetes mellitus (DM) is a highly prevalent metabolic disease with an increasing incidence worldwide (Benjamin et al., 2017). Microvascular and peripheral vascular calcification are the leading causes of amputation in patients with diabetes. Vascular calcification (VC), maladjustment that causes arteriosclerosis and dysfunction, is a severe complication. It is one of the key factors leading to type 2 diabetes mellitus (T2DM) (Harper et al., 2016; Lino et al., 2018). T2DM is closely linked with VC through several different mechanisms, some of which include oxidative stress, hyperglycemia, hyperkalemia, and apoptosis (Kay et al., 2016; Mozaffarian et al., 2016; Yahagi et al., 2017)

The advanced glycation end products (AGEs) are formed by Maillard reactions. They are proteins or lipids that become glycated nonenzymatically by glucose or other reducing sugars. Their derivatives include glyceraldehyde, glycolaldehyde, methylglyoxal, and acetaldehyde (Goldin et al., 2006). The expression of AGEs in patients with DM is increased, resulting in the accumulate extensively of AGEs in different apparatus, including blood vessels (Giacco and Brownlee 2010). Clinical studies have confirmed that AGEs induce allergic reactions and oxidative stress responses. These res*ponses occur via* the mediation of AGEs receptors (RAGE), which are major causes of vascular lesions. Apoptosis is also closely related to the calcification of smooth muscle cells (SMC). Previous studies have found that SMCs can release apoptotic bodies similar to matrix vesicles and induce further calcium deposition (Song et al., 2011). Sayo Koike believed that oxidative stress induced by NAD (P) H oxidase is related to AGEs/RAGE-induced apoptosis of vascular smooth muscle cells (VSMCs) (Koike et al., 2016). The interaction between AGEs and RAGEs may also hamper the normal functioning of endothelial cells constituting small arteries. This leads to the upregulated expression of various cytokines. Such as p38/MAPK and of wnt/β-catenin signaling pathway molecules activate SMC to accelerate atherosclerosis in patients with DM (Gudmundsson et al., 2016). Our previous study has demonstrated that AGEs/RAGE promotes Wnt/β-catenin axis-mediated calcification in HASMCs cells (Liu J. et al., 2016). In addition, direct *in vitro* induction of calcium (Ca^2+^) deposition by AGEs in the vascular smooth muscle cell line A7r5 has been reported with the involvement of oxidative stress mechanism. The oxidative stress mechanism is induced by NAD (P) H oxidase; apoptosis induced by oxidative stress was found to be one of the calcification mechanisms. These findings provide a basis for understanding the relationship between apoptosis and VC in DM (Koike et al., 2016).

Apart from the induction of VC, the role of AGEs-induced autophagy and apoptosis in DM has been reported. For example, upregulation of the COX-2/PGE2 pathway activated by NF-κB can further induce endothelial cell apoptosis by binding AGEs to its specific receptor RAGE (Lan et al., 2015). Additionally, Liu et al. have demonstrated that a high glucose environment can lead to the accumulation of AGEs (Liu Y. et al., 2016) and activate the caspase-3 signaling pathway, further inducing the apoptosis of rat osteoblasts. On the other hand, AGEs play a crucial role in suppressing podocyte autophagy under diabetic conditions (Zhao et al., 2018). However, the details of the mechanism underlying AGEs-induced autophagy and apoptosis and the molecular link between the two processes in calcification of human VSMCs have yet to be clarified (Garcia-Prat et al., 2016; Li et al., 2016).

Accordingly, the current study aimed to verify the effects of autophagy and apoptosis on calcification progression in VSMCs upon AGEs exposure. In particular, HASMCs were used as the cellular model because aortic atherosclerosis is one of the major vascular complications associated with DM (Liao et al., 2015). Besides, apoptosis inhibition and repression of VSMC-to-osteoblast transition were observed in AGEs treated autophagy-downregulated HASMCs. It has been noticed that Atg7 (autophagy-related gene 7) has defected, or autophagy inhibitor wortmannin (WM) was pretreated. Therefore, our findings suggest that autophagy, apoptosis, and the crosstalk between these processes participate in the mechanisms underlying the calcification of VSMCs. Compounds targeting the autophagic process induced by AGEs in VSMCs may serve as potential therapeutic agents for alleviating vascular complications associated with DM.

## Materials and Methods

### Cell Cultures

Human aortic smooth muscle cells (HASMCs) were purchased from ScienCell Research-Laboratory (United States). HASMCs Atg7 deficient cells were provided by Invitrogen Technology Co, Ltd (United States). Medium, FBS, and PSG were supplied by Gibco (Waltham, MA, United States). The incubator for cell-cultured was kept at 37°C with 5% humidified CO2.

### Experimental Reagents and Instruments

The specific concentrations of the reagents are in the text. The von Kossa Staining Kit (GMS80045.3), WM, and AGEs were purchased from GENMED Pharmaceutical Technology Co. Ltd. (Shanghai, China), Sigma (St. Louis, United States), and BioVision (San Francisco Bay Area, United States), respectively. Antibodies against ATG7 (2631), p62 (39786), caspase3 (9662), mTOR (2972), Bak(3814), caspase9 (9504), Bax (2772), p-mTOR (2971), LC3-II (2775), Beclin-1 (3495), and β-catenin (8480) were purchased from CST (Danvers, United States). Other antibodies against MMP7 (AP6212a), OPN (AP11567a), BMP2 (AP13858c), RAGE (AP6910c), and Runx2 (AP7735d) were acquired from Abgent (Nanjing, China). Antibodies against β-actin (sc-47778), α-SMA (906401), and OPG(GTX127948) were obtained from Gene Tex (Texas, United States), Bio legend (Peking, China), and Santa Cruz (MO, United States), respectively. HRP-, ZyMax^TM^ FITC-, and TRITC-conjugated secondary antibodies were purchased from Cell Signaling Technologies (United States) and Invitrogen (United States).

### Western Blotting

After 24 h of treatment, cell lysate (CST, United States) was collected and quantified with protein assay buffer (Bio-Rad, United States). SDS/PAGE separated proteins were transferred to PVDF films (GE, United Kingdom). Proteins binned with primary and secondary antibodies were detected by AI 600 (GE Healthcare, United Kingdom). Software Image J (NIH, United States) was used to measure the grayscale band strength normalized to beta-actin.

### Von Kossa Staining

HASMCs in the logarithmic phase were inoculated on glass slides, then 14-days AGEs treatment was done for all groups with medium-changing every 3 days. Each experimental group was set up with six parallel control groups. After the slides were removed, cells were washed with a clean solution, followed by 4% paraformaldehyde. Then, 200 μl staining solution was added to the cells, followed by sunlight exposure until deposited black particles were observed. After the staining solution removing, cells were rinsed with CenMed cleaning buffer and captured as images immediately.

### Flow Cytometry Analysis for Apoptosis

Cell toxicity assay was performed with BD flow cytometer following the standard instruction. Cells were cultured overnight and treated with the tested compounds for 72 h. Subsequently, trypsin and ice-cold PBS were used to detach and watch cells, respectively. Cells were stained with propidium iodide (50 μg/ml) and annexin V-FITC (2.5 μg/ml) in 100 μl buffer for 15 min in the dark. Ultimately, samples diluted with 300 μl binding buffer were measured by flow cytometer (BD FACSAria III, United States).

### Immunofluorescence Detection

A total of 2 × 10^5^ cells were cultured in a 6-well glass-bottomed plate. Processing cells on cover slides and treating for 24 h. Fixation with 4% paraformaldehyde, wash with PBS and permeabilize with methanol. Slides were incubated with target antibodies at 4°C for 12 h. Then, slides were incubated with TRITC anti-mouse antibody [1:200] at 37°C for 1 h after washing, and DAPI (Beyotime Biotechnology) for 15 min in the dark. Finally, using FluorSave™ medium (Calbiochem, United States), the dried slides were mounted onto the micro slide. Images were captured with Photometrics CoolSNAP HQ2 CCD camera on the Olympus IX71-Applied Precision Delta-Vision restoration microscope (Applied Precision, United States).

### Transmission Electron Microscopy

Electron microscopy monitors the autophagic flux process. Cell pellets were collected and fixed in 0.1 M phosphate buffer for 45 min with 2.5% glutaraldehyde, post-fixed for 1 h in 1% OsO4, alcohol dehydrated, and embedded in Araldite. Slides were stained by uranyl acetate with lead citrate and analyzed on a Philips CM10 transmission electron microscope.

### F-Actin Staining

Cells (2 × 10^5^ cells/well) were seeded in a 6-well plate and incubated overnight. Then cells were treated with the tested compounds for 72 h. After washing with ice-cold PBS, cells were stained with DAPI (Beyotime, United States) and rhodamine-phalloidin (1:200, Sigma, MO, United States) to mark the cytoblast and cytoskeleton, respectively. Pictures were acquired by Photometrics CoolSNAPHQ2 CCD camera on the Olympus IX71-Applied Precision Delta Vision restoration microscope (Applied Precision, United States) and deconvolved using Delta Vision algorithms (Applied Precision, United States).

### Intracellular Ca^2+^ Measurement

Intracellular cytosolic Ca^2+^ was determined with FLIPR Calcium 6 Assay Kit (Molecular Devices, United States). Simultaneously, cells were treated for 30 min with tested compounds and followed by observation under the fluorescence microscope. Green fluorescence indicates the area where calcium deposits.

### Animal Experiments

Mice were intraperitoneally injected with a dose of 160 mg/kg STZ (Zanosar Teva pharmaceuticals, Irvine, CA). Blood for glucose concentration monitoring was obtained from the tail. Blood glucose concentration and was monitored daily before and after the injection of STZ. Mice with a glucose concentration exceeding 300 mg/dl are considered to be diabetes. Wortmannin was given once/2 days for 20 days at a dosage of 0.3 mg/kg after the diabetic model was established. Aorta samples were collected, fixed with 4% paraformaldehyde for 48 h, processed with paraffin, and cut into sections at 5 μm thickness. HE-stained sections were examined by microscope for vessel calcification evaluation. Immunohistochemistry detection for RUNX2 and LC3-II has been performed on the Dako Link 48 System. Antigen retrieval was completed using Dako Target Retrieval Solution in low pH (pH6.1) at 97°C for 20 min. Antibody was diluted in 1:4000, and the protein signal was detected by Dako Flex + (Polymer system) with DAB as the chromogen.

### Statistical Analysis

Data of western blot are presented as the fold change compared with the control group. Each point represents the mean ± SD (*n* = 3/group), *t*-test. Quantification of apoptotic ratio in flow cytometry analysis, immunofluorescence for LC3-II autophagic puncta counting, and von Kossa staining of calcification detection were analyzed mean value ± S.D, one-way ANOVA. All statistical analysis and artwork plotting were done basing on GraphPad 8.0. *p* < 0.05 was considered statistically significant. *p < 0.05, **p < 0.01, ***p < 0.001; ^#^ *p < 0.05,* ^##^ *p < 0.01,* ^*###*^ *p < 0.001.*

## Results

### Advanced Glycation End Products Induce Calcification *via* the Wnt/β-Catenin Axis in Human Aortic Smooth Muscle Cells

**FIGURE 1:**
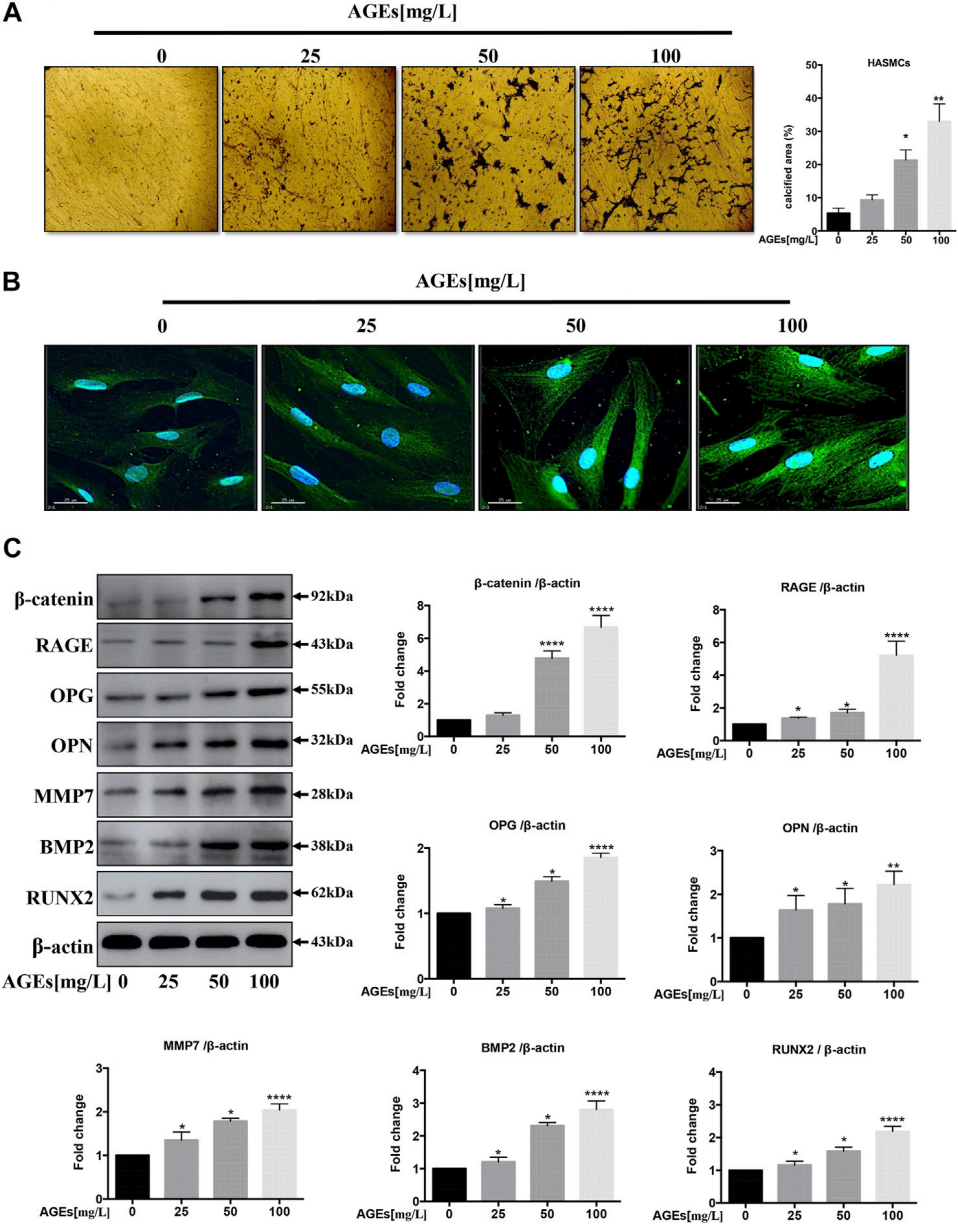
AGEs induce calcification in HASMCs. **(A)** Dose-dependent study of AGEs-induced calcification in HASMCs at AGEs concentrations from 0 to 100 mg/L. Von Kossa staining (black) in HASMCs after AGEs treatment for 2 weeks. scale bar = 100 µm (×10). **(B)** Immunofluorescence staining of calcification-related proteins with 24 h’ AGEs treatment at indicated concentrations. The green GFP signal represents the calcified protein OPN. The blue DAPI signal represents the nuclear region, scale bar, 25 µm. **(C)** Western blot analysis of the expression of RAGE and other calcification-related proteins (β-catenin, OPG, OPN, MMP7, BMP2, Runx2) with 24 h’ treatment. Data are presented as the fold change compared with the control group. Each point represents the mean ± SD (*n* = 3/group); ^*^ *p* < 0.05, ^**^ *p* < 0.01, ^***^ *p* < 0.001.

AGEs are capable of inducing Ca^2+^ deposition in a rat VSMC line (Zhu et al., 2018). Since the effect of AGEs is strongly dependent on their preparation procedure, we first determined whether the AGEs used in this study could also induce the reported effect in HASMCs. First, Von Kossa staining was adopted to monitor the Ca^2+^ deposition in AGEs-treated HASMCs. As demonstrated in Figure 1A, the administration of AGEs induced Ca^2+^ deposition (black deposits) in our cellular model in a dose-dependent manner, within the range of 25 mg/L to 100 mg/L compared to no AGEs treatment. The concentrations used were based on the preliminary experiments (Yuan et al., 2020) that stimulate calcification in HVSMC in a dose-dependent manner *in vitro* (Kay et al., 2016). Sayo Koike's study produced the same results (Koike et al., 2016). We further analyzed the effects of AGEs treatment on the transition of HASMCs to osteoblasts by comparing the cells to untreated control cells. Immunofluorescence staining was used to visualize the expression of the osteoblast marker osteopontin (OPN). The expression of OPN (green) in HASMCs (Figure 1B) was induced in a dose-dependent manner at the same range of AGEs concentrations. This was concordant with the results acquired by Western blot analysis (Figure 1C). Cai et al. claimed that the Wnt/β-catenin pathway could directly stimulate osteogenic calcification accompanied with differentiation in VSMCs via Runx2 regulation (Cai et al., 2016). In addition to OPN, RAGE and a panel of calcification-related proteins that are downstream genes of Wnt/β-catenin were upregulated. These genes are matrix metalloproteinase 7 (MMP7), β-catenin, osteoprotegerin (OPG), bone morphogenetic protein 2 (BMP2), and runt-related transcription factor (Runx2) (Albanese et al., 2018). As demonstrated by the Western blot results in Figure 1C, the effects were correlated with the concentration of AGEs administered. Therefore, the AGEs employed in this study effectively induced Ca^2+^ deposition in aortic SMC to facilitate their transition into osteoblast-like cells representing the initial stage of atherogenesis.

### Advanced Glycation End Products Induce mTOR-Mediated Autophagy in Human Aortic Smooth Muscle Cells

**FIGURE 2:**
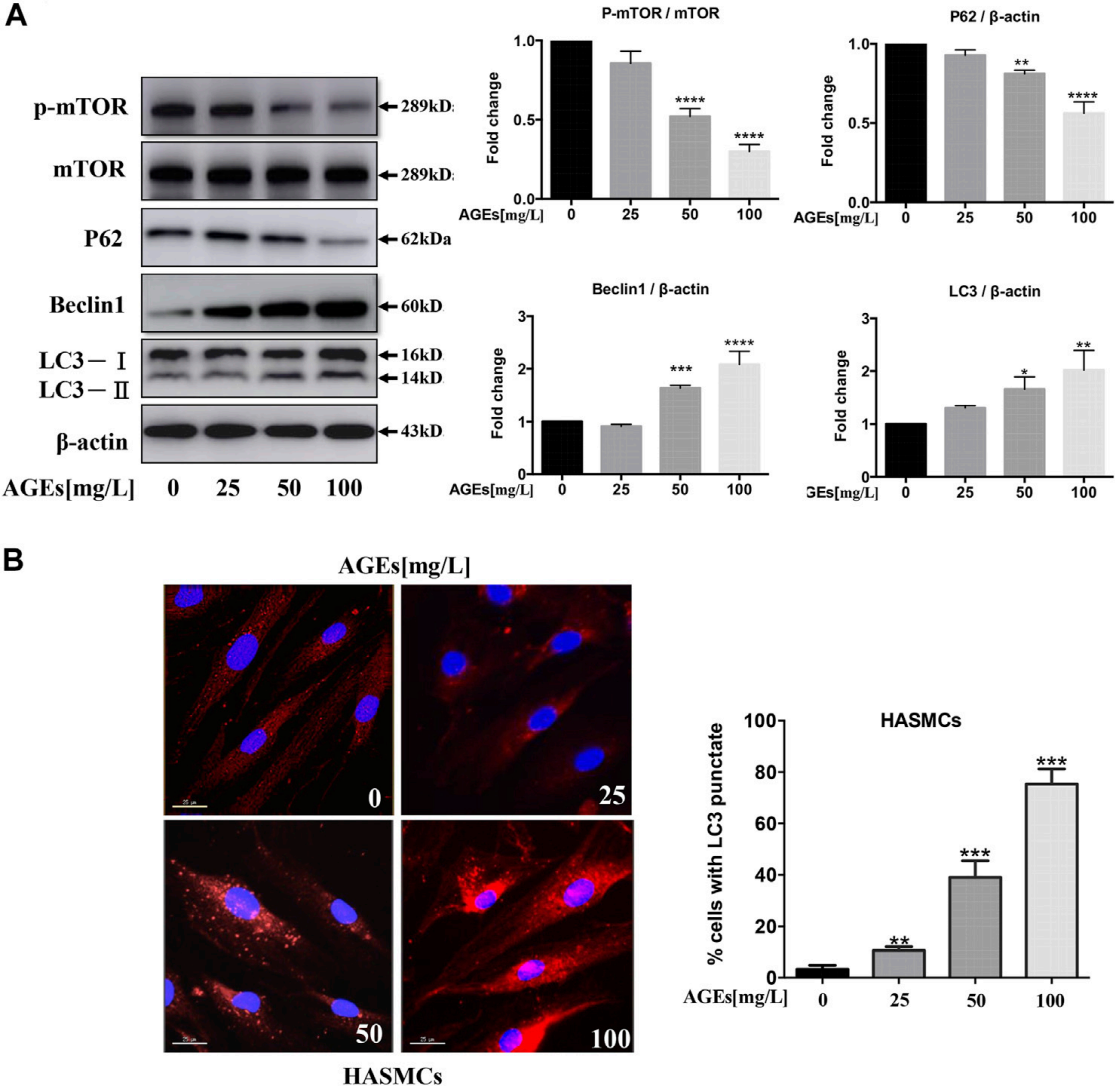
AGEs induce autophagy *via* mTOR signaling in HASMCs. Dose-dependent study of AGEs-induced autophagy in HASMCs at AGEs concentrations from 0 to 100 mg/L with 24 h’ treatment. **(A)** Western blot analysis of mTOR and autophagy-related proteins (p62, beclin1, and LC3) in AGEs-treated HASMCs. **(B)** Immunofluorescence analysis of LC3-II puncta formation (red color represents LC3-II, blue represents DAPI; scale bar = 25 μm). Data are presented as the fold change compared with the control group. Each point represents the mean ± SD (*n* = 3/group); ^*^ *p* < 0.05, ^**^ *p* < 0.01, ^***^ *p* < 0.001.

Previous studies have found that AGEs can induce autophagy in cardiomyocytes (Hu et al., 2015). To determine whether autophagy could also be induced by AGEs treatment in HASMC, we cultured SMC with different concentrations of AGEs of 0–100 mg/L for 24 h. Firstly, autophagy biomarkers were detected and normalized to DMSO-treated control. It was demonstrated that AGEs could increase the expression level of LC3-II and beclin1 in a dose-dependent manner, while p-mTOR and P62 shown contrary tendencies (Figure 2A). The result showed that AGEs stimulated autophagy *via* an mTOR-related pathway in HASMCs. Besides, it was further proved that AGEs dose-dependently increase endogenies LC3 puncta formation around the cell membrane of HASMCs by immunofluorescence detection (Figure 2B).

### Advanced Glycation End Products Induce Apoptosis in Human Aortic Smooth Muscle Cells

**FIGURE 3:**
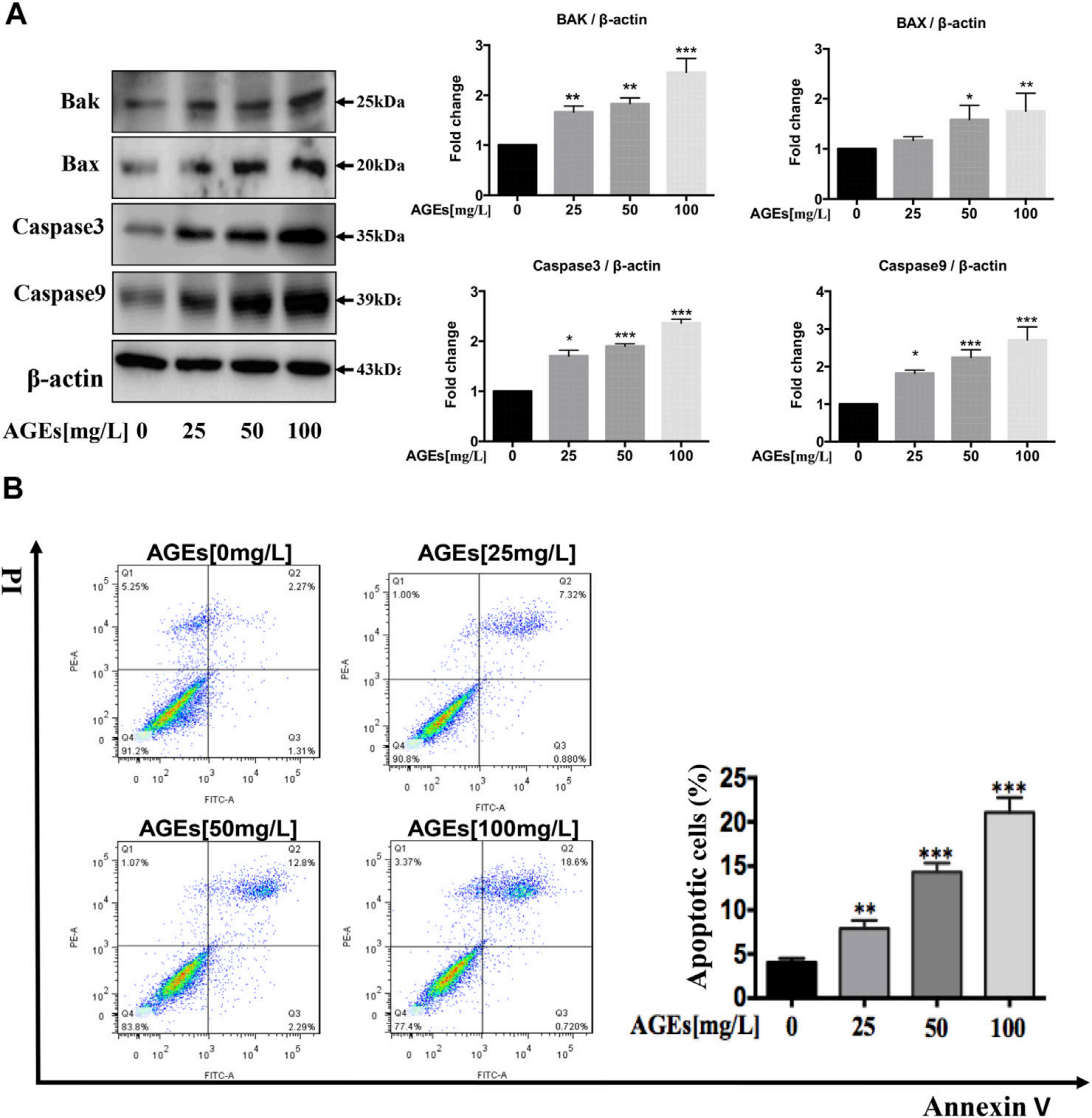
AGEs induce apoptosis in HASMCs. Dose-dependent study of AGEs-induced apoptosis in HASMCs at AGEs concentrations from 0 to 100 mg/L. **(A)** Western blot analysis of apoptosis-related proteins (Bak, Bax, caspase-3, and caspase-9) in AGEs-treated HASMCs. **(B)** Flow cytometry analysis of apoptosis in AGEs-treated HASMCs. Data are presented with mean percentages of Q2 and Q3 quadrant. Each bar represents the mean ± SD (*n* = 3/group); ^*^ *p* < 0.05, ^**^ *p* < 0.01, ^***^ *p* < 0.001.

HASMCs were incubated with AGEs at concentrations ranging from 0 to 100 mg/L for 24 h. Cysteine proteases caspase-3 and caspase-9 are necessary for activating the cascade reaction during apoptosis and have been confirmed to be crucial to apoptosis and necrosis. Bax and Bak are pro-apoptotic proteins whose expression is positively correlated with apoptosis. To identify if AGEs could induce apoptosis, Bak and Bax and the cleaved forms of caspase-3 and caspase-9 were detected in HASMCs. The results indicated that AGEs induced the expression of the apoptosis-related proteins Bax, Bak, caspase-3, and caspase-9 in a dose-dependent manner, as shown in Figure 3A. Besides, Annexin V-PI-stained flow cytometry assay confirmed that apoptosis was significantly stimulated after 24 h’ treatment by AGEs (Figure 3B). Consistently, AGEs can also increase apoptosis in a concentration-dependent manner (Figure 3B). These results demonstrated that AGEs induced apoptosis in this cell type. As reported by Marino et al., autophagy and apoptosis have shown synchronicity, in which autophagy generally precedes apoptosis (Marino et al., 2014). We showed in our study that AGEs could induce not only autophagy but also apoptosis in HASMCs.

### Effects of an Autophagy Inhibitor on Advanced Glycation End Products-Induced Autophagy and Apoptosis in Human Aortic Smooth Muscle Cells

**FIGURE 4:**
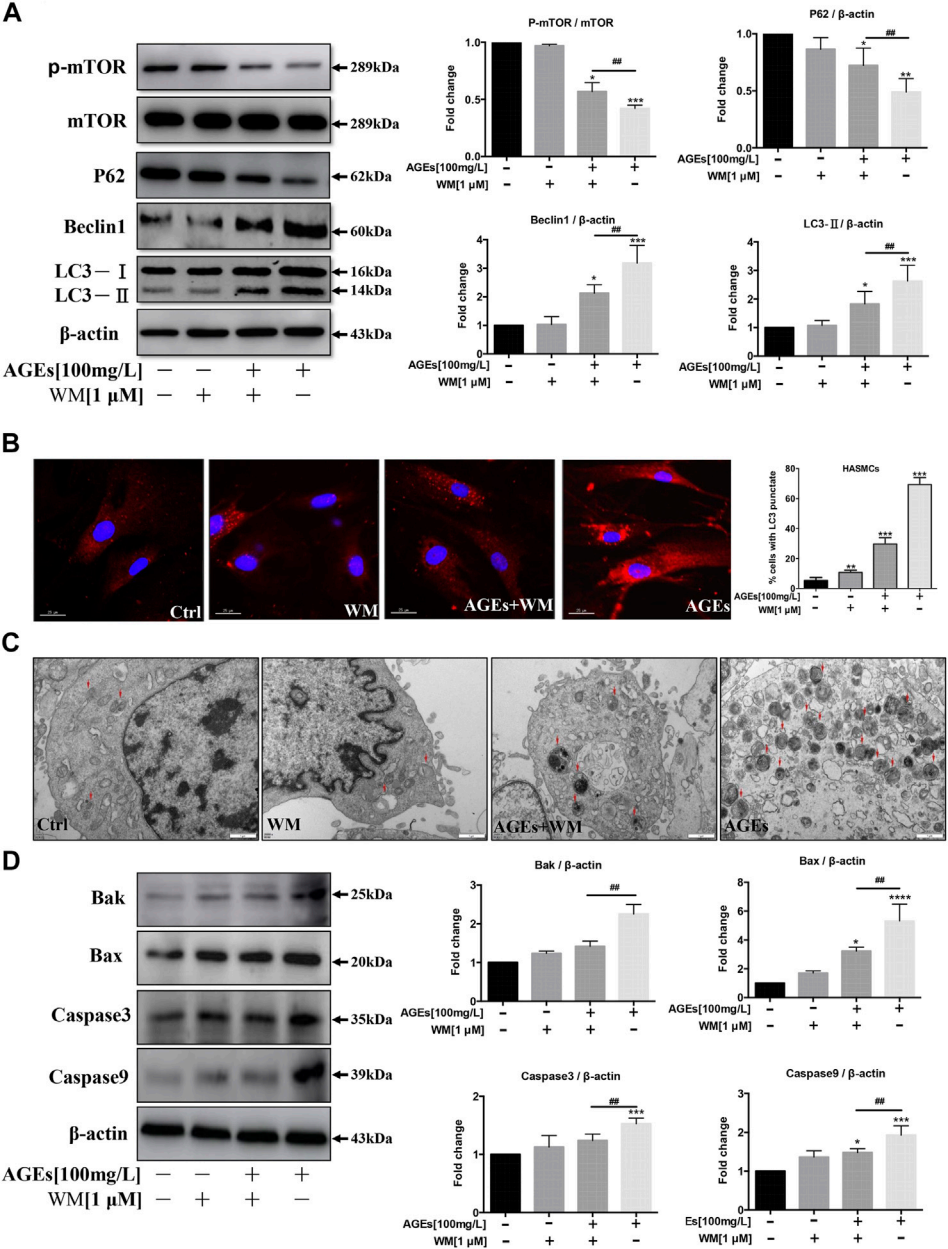
Effects of an autophagy inhibitor on autophagy and apoptosis in HASMCs. After pretreatment with wortmannin (WM) for 1 h, HASMCs were incubated with AGEs (100 µg/ml) for another 24 h **(A)** WM suppressed AGEs-induced autophagy marker expression in HASMCs. Western blot analysis of autophagy proteins (LC3, beclin1, p62, and p-mTOR) in HASMCs treated with WM and AGEs for 24 h **(B)** WM abolished AGEs-induced autophagic puncta formation in HASMCs. Immunofluorescence analysis of LC3-II puncta formation (red color represents LC3-II, blue represents DAPI; scale bar = 25 μm). **(C)** TEM evidence of autophagic vacuoles (red arrows), bars: 1 μm. **(D)** Western bDlot analysis of apoptosis-related proteins (Bak, Bax, caspase-3, and caspase-9) in WM/AGEs -treated HASMCs. Data are presented as fold change compared with the control group. Each point represents the mean ± SD (*n* = 3/group); ^*^ *p* < 0.05, ^**^ *p* < 0.01, ^***^ *p* < 0.001 compared with the WM/AGEs-treated group; ^*#*^ *p* < 0.05, ^##^ *p* < 0.01, ^###^ *p* < 0.001.

We investigated whether wortmannin (WM)-mediated autophagy inhibition is capable of preventing AGEs-induced autophagy and apoptosis in HASMCs. We, therefore, blocked the autophagic activity of AGEs by using WM, an inhibitor of autophagy. HASMCs were pretreated with WM (1 µM) for 1 h before the addition of AGEs. Correspondingly, WM participation decreased LC3-II and Beclin-1 expressions, which were highly expressed after AGEs treatment. Simultaneously, p-mTOR and P62 levels showed the opposite tendency (Figure 4A). This demonstrated that WM could inhibit AGEs-induced autophagy. As observed in Figure 2B, the immunofluorescence revealed that in comparison to controls, AGEs increased the intracellular localization of red LC3 puncta. As expected, WM pretreatment caused less LC3 puncta formation in AGEs-stimulated cells. The addition of WM markedly suppressed the AGEs-mediated accumulation of autophagosomes, indicating that autophagy activity was significantly inhibited. Consistently, LC3-II in HASMCs significantly decreased under WM and AGEs co-treated compared with AGEs single treatment (Figure 4B). In Figure 4C, the gold standard for autophagy flux detection, transmission electron microscopy (TEM), was performed in HASMCs. Autophagic vacuoles (marked with red arrow) stimulated by AGEs were markedly suppressed when co-treating with WM. Nevertheless, the apoptosis biomarkers, like Bax, Bak, caspase-3, and caspase-9, were significantly decreased in WM-pretreated AGEs-treated cells (Figure 4D). These results demonstrate that WM can inhibit AGEs-induced apoptosis in HASMCs.

### WM Suppresses Phenotypic Switching in Advanced Glycation End Products-Treated Human Aortic Smooth Muscle Cells

**FIGURE 5:**
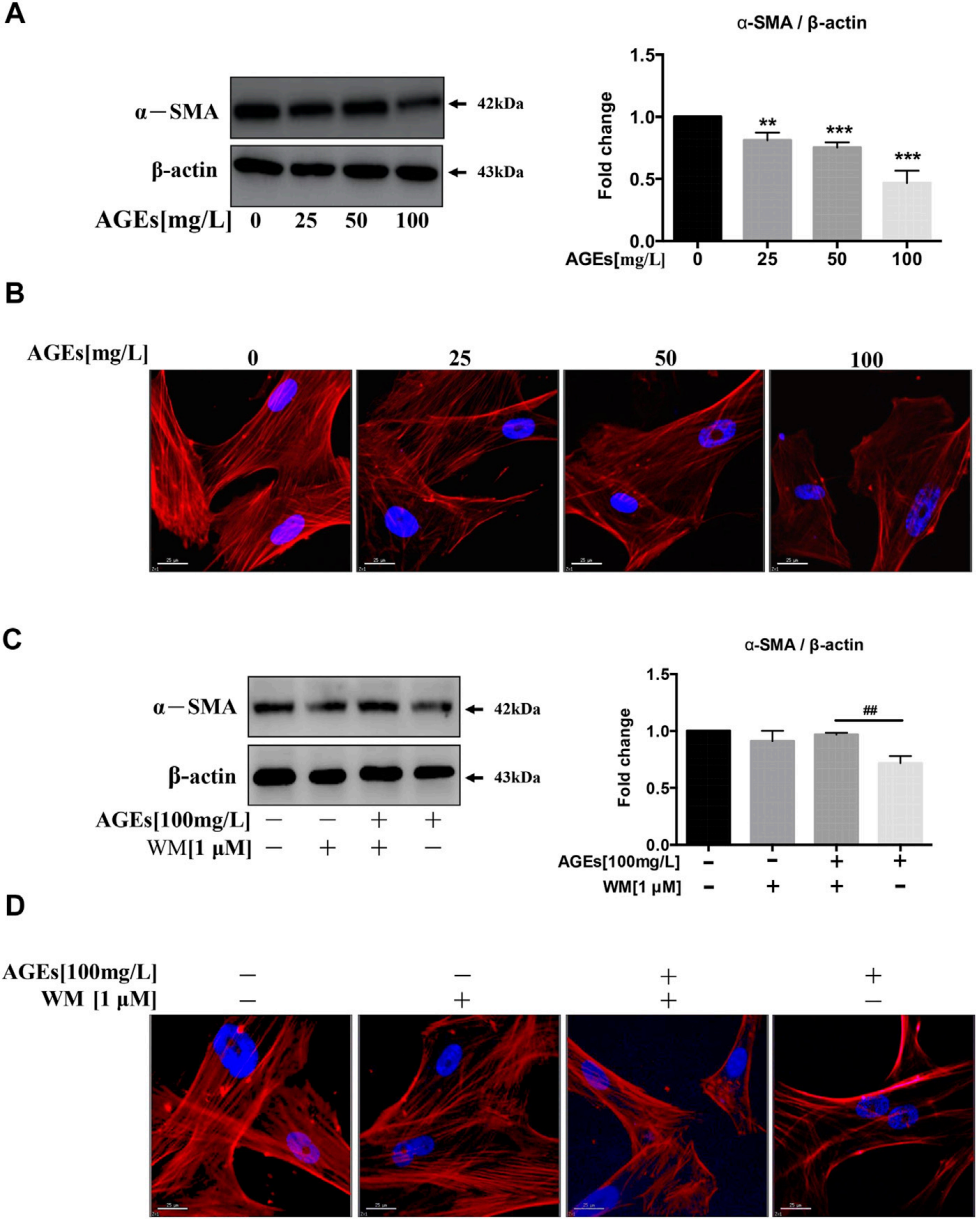
WM suppresses phenotypic switching in AGEs-treated HASMCs. Cells were treated with AGEs for 24 h. **(A)** Western blot analysis of α-SMA (a smooth muscle cell-specific protein). **(B)** Observation of the cell cytoskeleton in HASMCs after treatment with AGEs (0–100 mg/L) (red represents F-actin, blue represents DAPI; scale bar = 25 μm). **(C)** Western blot analysis shows the levels of α-SMA after WM administration (1 µM) in HASMCs. **(D)** Cytoskeletal staining assay verifies osteoblast phenotype (red represents F-actin, blue represents DAPI; scale bar = 25 μm). Data are presented as the fold change compared with the control group. Each point represents the mean ± SD (*n* = 3/group); *p < 0.05, **p < 0.01, ***p < 0.001 compared with the WM/AGEs group; ^*##*^ *p* < 0.01.

VC shows an analogical process with bone formation (Giachelli 2004). SMC functions can be altered from contraction to synthesis during calcification. At the same time, they demonstrate low expression of SMC-specific contractile markers, such as α-SMA, SM22α, and calponin (Chen et al., 2015). It was found that AGEs can decrease the level of α-SMA, a smooth muscle cell-specific protein, in a dose-dependent manner (Figure 5A). We further identified that F-actin protein is abundant in untreated HASMCs. At the same time, the expression level of F-actin was inhibited by AGEs treatment, which also disrupted the intercellular actin cytoskeletal structure (Figure 5B). Collectively, the trans-differentiation of SMCs into osteoblasts-like cells is inhibited by AGEs.

We next investigated whether WM-abolished AGEs-induced autophagy participated in the phenotypic transition process of SMCs. We incubated AGEs (100 mg/L) in HASMCs in the presence or absence of the WM (1 µM), the autophagy inhibitor. The results showed that inhibition of AGEs-induced autophagy suppressed AGEs-mediated downregulation of α-SMA (Figure 5C). Simultaneously, AGEs-treated cells with 2-h WM pretreatment showed a higher expression level of F-actin protein (Figure 5D). This demonstrates that AGEs-stimulated autophagy could promote the differentiation process from HASMCs to osteoblasts.

Furthermore, the high level of F-actin observed in control cells showed a decrease in AGEs-treated HASMCs. WM treatment improved or restored this decrease in F-actin expression caused by AGEs. Thus, the inhibition of the phenotypic transition process from SMC to osteoblasts was demonstrated.

### Wortmannin Inhibits Calcification in Human Aortic Smooth Muscle Cells by Inhibiting Autophagy

**FIGURE 6:**
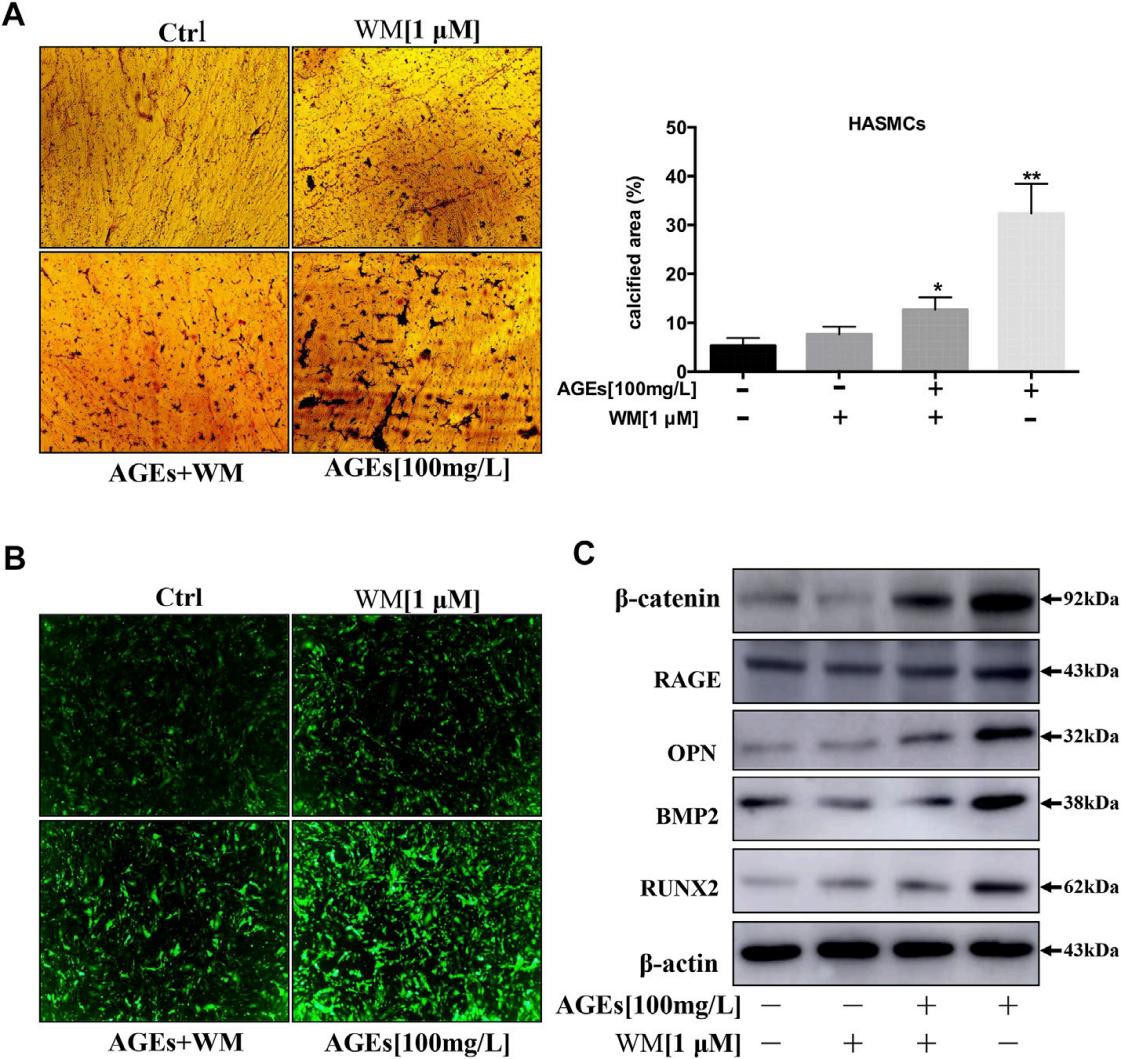
WM inhibits the calcification of HASMCs by inhibiting autophagy. **(A)** Deposition of calcium was quantified by von Kossa staining in 14 days-AGEs and WM-treated HASMCs. The black spots in the image represent calcified areas; scale bar = 100 µm (×10). Each point represents the mean ± SD (*n* = 3/group). **(B)** Intracellular Ca^2+^ was quantified with FLIPR Calcium 6 dye (green) in AGEs and WM-treated HASMCs; scale bar = 100 µm (×10). **(C)** Western blot analysis of calcification-related proteins (β-catenin, RAGE, OPN, BMP2, and Runx2) in HASMCs.

HASMCs cotreated with WM and AGEs (100 µg/ml) showed lower levels of autophagy, and WM inhibited AGEs-induced apoptosis in HASMCs. Thus, we determined whether WM could inhibit AGEs-induced VC. In Figure 6A, the process of calcification showed a significant increase in HASMCs when treated with AGEs. At the same time, WM existence attenuated the effect of AGEs-stimulated calcification. A plate with von Kossa staining showed that administration of WM in AGEs-treated cells resulted in a reduction of black spots both in the nucleus and cytoplasm (Figure 6A). Furthermore, the amount of intracellular calcium was visualized via the FLIPR-calcium-6 kit in HASMCs. The GFP-Ca^2+^ increased noticeably in AGEs-induced cells. In line with previous results, WM significantly suppressed the GFP-Ca^2+^ signal in AGEs-stimulated HASMCs (Figure 6B).

At present, some studies investigated that beneficial autophagy could protect the stability of atherosclerotic plaque. However, excessive autophagy can further aggravate atherosclerosis (Mialet-Perez and Vindis 2017). Besides, the expression of calcification-related proteins such as β-catenin, RAGE, Runx2, OPN, and BMP2 was noticeably suppressed by WM, the autophagy inhibitor (Figure 6C). Thus, our findings suggest that inhibition of autophagy can suppress apoptosis and calcification *via* the inhibition of Wnt/β-catenin signaling pathways. Hence, AGEs were verified to induce calcification in HASMCs *via* the induction of autophagy.

### Effects of Atg7 on Autophagy, Apoptosis, and Calcification in Human Aortic Smooth Muscle Cells

**FIGURE 7:**
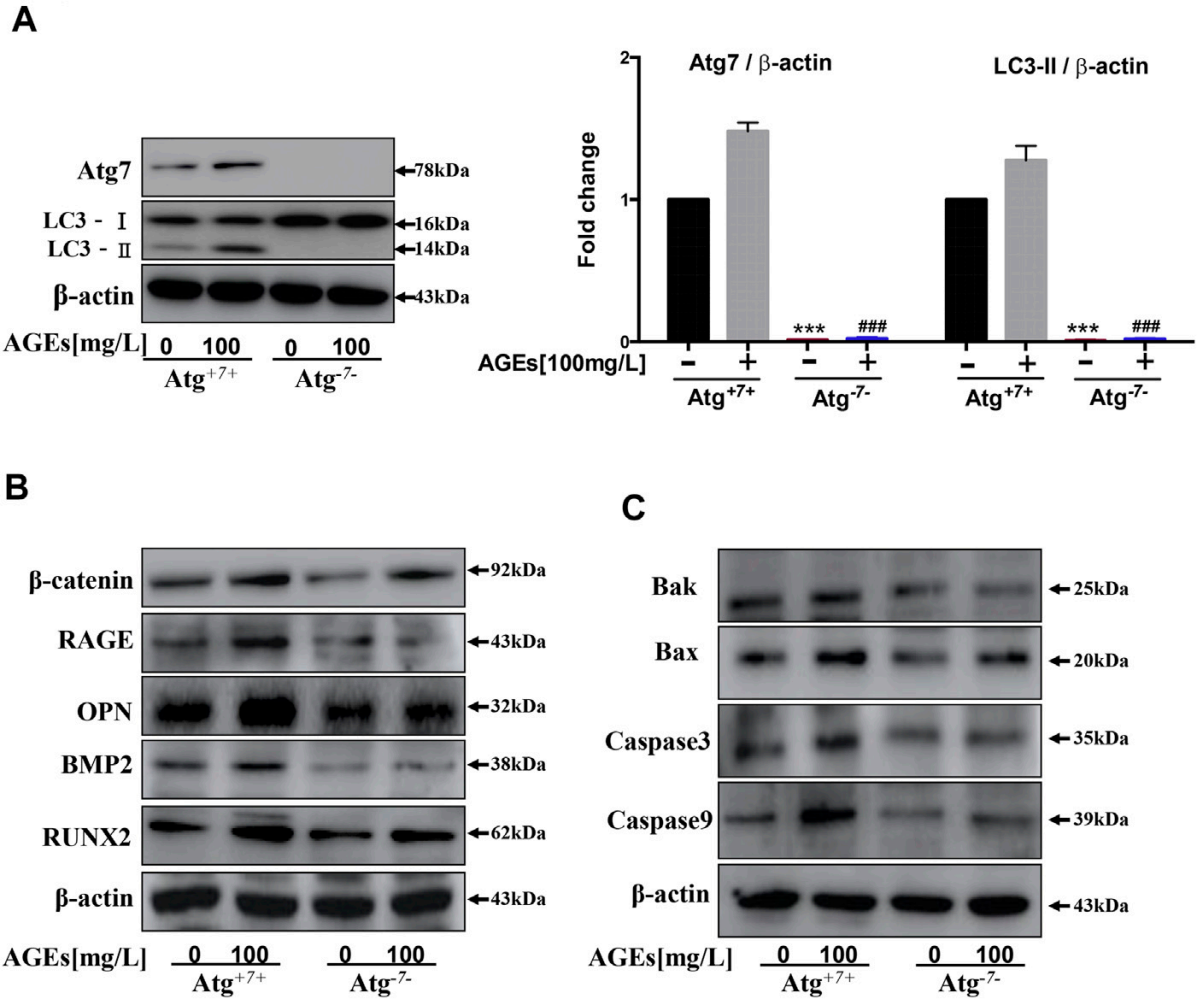
Atg7 knockout can reduce AGEs-induced apoptosis and calcification. Cells were treated with AGEs for 24 h. **(A)** Changes in protein expression in Atg7 knockout HASMCs. Western blot analysis of LC3 and Atg7. **(B)** Western blot analysis of calcification-related proteins (β-catenin, Runx2, OPN, and BMP2) in AGEs-treated HASMCs with or without Atg7 knockout. **(C)** Western blot analysis of apoptosis-related proteins (Caspase3, Caspase9, Bax, and Bak) in AGEs-treated HASMCs with or without Atg7 knockout. Each point represents the mean ± SD (*n* = 3/group). ^***^ *p < 0.001* and ^###^ *p < 0.001* versus Atg7 and LC3 expression level in WT cells respectively, *t*-test analysis.

Atg7 is an upstream gene of LC3 that is essential to convert LC3-I to LC3-II in the autophagy process (Deshpande et al., 2018). Atg3 and Atg7 interaction is required for lipidation and membrane transfer of LC3. This could be suppressed by enzymatic oxidation (Kaiser et al., 2012). Thus, levels of Atg7 in AGEs-induced autophagy were detected. In Figure 7A, the knockout of Atg7 in HASMCs showed abolishment in LC3-I to LC3-II transformation. This indicated that Atg7 is indispensable in AGEs-induced autophagy. Furthermore, the role of Atg7 in apoptosis and calcification of HASMCs induced by AGEs were detected. The results in Figure 7B showed that the level of calcification-related proteins BMP2, Runx2, OPN, and β-catenin showed suppression in Atg7 knockout cells. This suppression is parallel with the significant decrease of RAGE, the AGEs receptor, and the expression of apoptosis-related proteins such as caspase9, caspase3, Bax, and Bak (Figure 7C). Collectively, these findings demonstrated that Atg7 knockout could rescue AGEs-stimulated calcification and apoptosis.

### Autophagy Inhibition Rescues *in vivo* Aortic Calcification in STZ Diabetic Mouse Model

**FIGURE 8:**
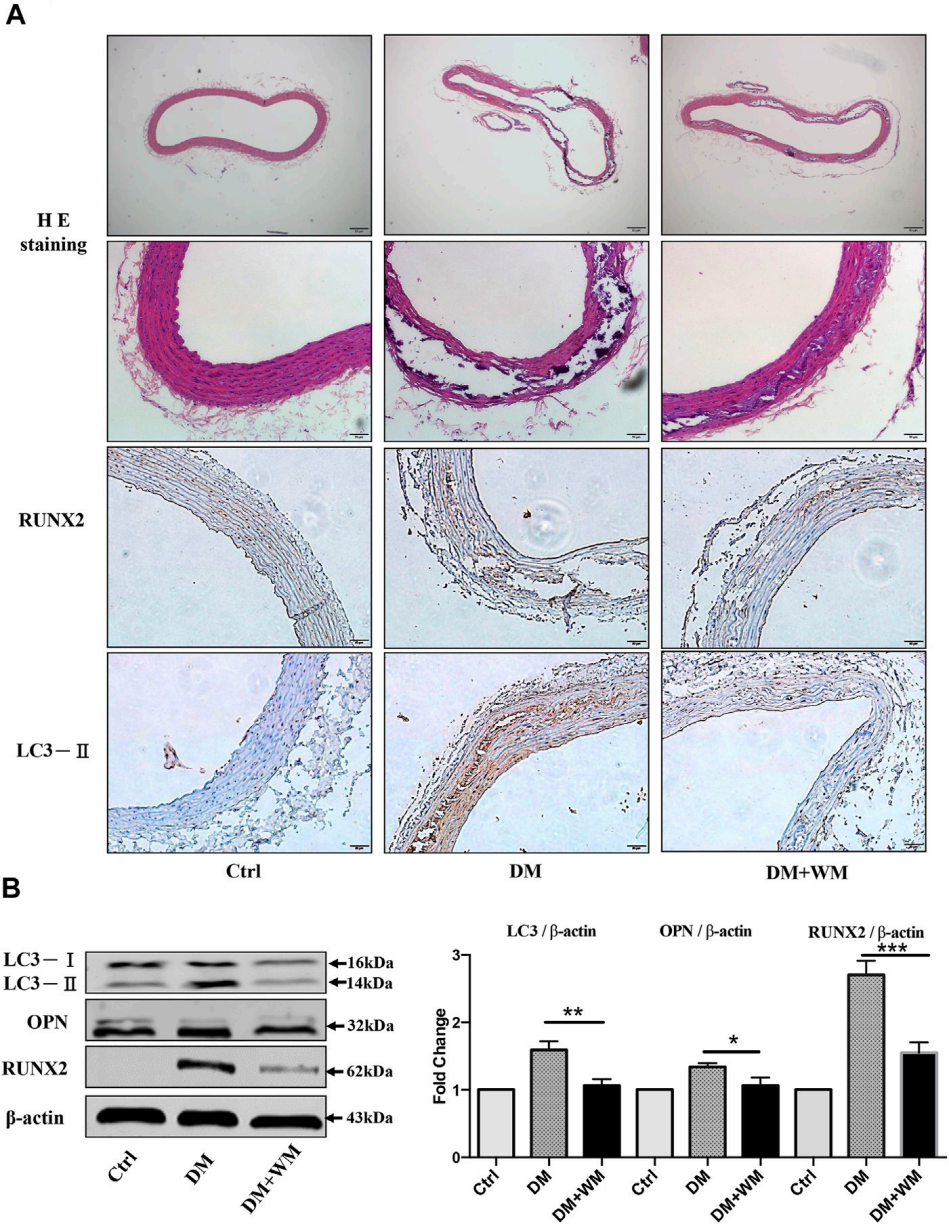
Autophagy suppression rescues aortic calcification *in vivo*. **(A)** Representative images of HE and immunohistochemistry-stained aorta sections are shown. Areas of calcification lesions are shown with black and white tissues in detailed HE images at higher magnification. Immunohistochemistry images stained with RUNX2, and LC3-II are shown in lower panels. Tissues with brown color stand for the positive signal for corresponding antibodies. Scale bar = 50 μm (×10), scale bar = 10 μm (×20). **(B)** Western blot assay from the homogenized samples from the same aorta sections was detected. The expression of LC3-II was significantly higher in the DM group than WM co-treated group. Besides, RUNX2 and OPN expression in aortic tissue significantly decreased than in the DM group.

In order to evaluate the effect of autophagy inhibitors on apoptosis, HE staining was performed on the STZ-induced diabetic atherosclerosis mouse model. This was done to assess the histopathology of aortic slices and the degree of VC. As the HE results are shown in Figure 8A, the diabetic mice (DM) have typical atherosclerotic pathological changes. These include thin fiber caps, foam cells, inflammatory cells, and cholesterol crystals in atherosclerotic plaques. In addition, after administration of the autophagy inhibitor, wortmannin (WM), the area of atherosclerotic plaques was significantly reduced. Then, immunohistochemical analysis was used to detect the expression of calcification-related protein RUNX2 in aortic slices. The results in the second-lowest panel of Figure 8A showed that RUNX was significantly highly expressed in atherosclerotic plaques in the DM group. At the same time, the expression level of RUNX2 in the aortic tissue of the WM treated DM group was markedly reduced.

Furthermore, the homogenized samples from the same aortas were detected by Western blot assay in Figure 8B. The expression level of autophagy-related protein LC3-II in the DM group was significantly higher than that of the WM co-treated group. Simultaneously, the expression level of calcification-related proteins RUNX2 and OPN in aortic tissue from WM-treated diabetic mice significantly decreased, comparing with DM alone. Collectively, autophagy suppression could markedly rescue the aortic calcification *in vivo*.

## Discussion

This study demonstrated that autophagy and apoptosis inhibition have potency to rescue HASMCs from AGEs-promoted calcification. The results showed that AGEs could induce apoptosis and calcification and the activation of Wnt/β-catenin signaling in HASMCs. In addition, AGEs activated autophagy, which could be attenuated by autophagy inhibitor treatment and Atg7 knockout. Therefore, we concluded that autophagy suppression protected HASMCs against apoptosis and calcification by inhibiting the Atg7/mTOR-dependent autophagy pathway and downregulating Wnt/β-catenin signaling molecules. This study did not determine the expression of alkaline phosphatase (ALP), an indicator of calcification, in AGEs- or WM-treated HASMCs. Collectively, drugs with an autophagy-suppressive effect may be used to prevent or inhibit VC. This provides new ideas on therapeutic strategies for the attenuation of VC. Based on our *in vitro* findings, it is worth investigating if AGEs treatment activates autophagy and accelerates VC in vascular calcification mouse models. Besides, autophagy status in VC in the patient with diabetes mellitus is worth investigating.

DM is an endocrine disease characterized by the interaction between genetic and environmental factors leading to hyperglycemia. According to investigations, China has the highest incidence rate of diabetes in the world (Guariguata et al., 2014). A series of interactions between elevated blood glucose levels and proteins occur in patients with DM, which eventually lead to the production of AGEs (Singh et al., 2014). After binding to their receptor, RAGE, the abnormally accumulated AGEs activate various signaling pathways *in vivo*. This promotes the differentiation of VSMCs into osteoblasts, eventually vascular calcified (Kapustin and Shanahan 2016; Kay et al., 2016; Wang et al., 2016). This study showed that the expression of calcification-related proteins and RAGE in HASMCs was significantly increased after AGEs intervention. Von Kossa staining also indicated that high quantities of calcium deposits formed in cells after AGEs intervention in a dose-dependent manner. This suggests that, in HASMCs, AGEs can significantly increase the expression of RAGE, activate various signaling pathways *in vivo*, and increase the expression of calcification-related proteins. Finally, these pathways result in the induction of calcification.

Apoptosis is one of the processes leading to VC. Extensive studies have confirmed that apoptosis is involved in VC(Koike et al., 2016). Cysteine proteases caspase-3 and caspase-9 are necessary for activating the signaling cascade in apoptosis and are thus crucial to apoptosis and necrosis (Redza-Dutordoir and Averill-Bates, 2016). Bax and Bak are pro-apoptotic proteins, and their expression is positively correlated with apoptosis (Bai et al., 2004; Peng et al., 2016). This study revealed that after HASMCs were treated with AGEs, the expression of caspase 9, caspase 3, Bax, and Bak was significantly increased in a concentration dependence. Furthermore, the greater the concentration of AGEs, the higher was the apoptotic rate in HASMCs, indicating that AGEs can induce HASMCs apoptosis, which is consistent with the report of Gao et al. (2015).

Autophagy, a pathway to programmed cell death different from apoptosis, is widely observed in various diseases (Furukawa et al., 2017). Studies have shown that autophagy can be detected in calcified SMC. Therefore, autophagy, similar to apoptosis, may involve VC (Grootaert et al., 2018). LC3 plays an essential role in the occurrence and development of autophagy. The synthesized carboxyl terminus of LC3 is rapidly lysed to produce cytoplasmic LC3-I. During the process of autophagy, cytoplasmic LC3-I is lipidated and converted to LC3-Ⅱ, thereby linking LC3 to autophagic vesicles. The presence of LC3 in autophagosomes and their conversion to the smaller migrating from LC3-II is typically used as an “indicator” of autophagy (Masuda et al., 2016). It has been suggested that beneficial autophagy can protect the stability of atherosclerotic plaque. However, excessive autophagy can further aggravate atherosclerosis (Marino et al., 2014). Our study demonstrated that AGE-induced autophagy might aggravate calcification of HASMCs, suggesting that AGE-induced autophagy is excessive. Our results showed that AGEs could induce not only autophagy but also calcification. To investigate the relationship between autophagy, calcification, and apoptosis in HASMCs, autophagy inhibitor WM was used. We found that WM treatment can reduce AGEs-induced autophagy and simultaneously attenuate calcification. Therefore, our results indicated that inhibition of AGEs-induced autophagy could prevent the apoptosis of HASMCs cells and the transformation of osteoblasts. We did not assess other pathways related to mTOR in AGEs or WM-treated HASMCs.

As an essential physiological response, autophagy establishes a different “dynamic balance” under different conditions in different forms, strictly regulated by the body, to maintain homeostasis (Liu et al., 2018). Basal levels of autophagy under physiological conditions maintain normal functions of the body (Feng et al., 2015). Cells under metabolic stress or threatened by cytotoxicity initiate autophagy, occurring at a low level, which drives the body into a pathophysiological state (Barlow and Thomas 2015). On the other hand, excessive autophagy results in autophagic cell death, causing damage to tissues and organs and impairs their physiological functions (Evangelisti et al., 2015). Therefore, “unbalanced” and “uncontrolled” autophagy can eventually cause disorders of the immune system, resulting in many diseases, including neurodegenerative diseases and tumors (Kiriyama and Nochi 2015). Wortmannin (WM) inhibits autophagy via suppression of the PI3K pathway. This study found that WM can effectively suppress AGEs-induced autophagy in HASMCs. In addition, once the WM was introduced, the level of cytosolic calcification biomarkers was decreased remarkably, as was the intracellular content of Ca^2+^. This suggests that the inhibition of autophagy can greatly inhibit calcification. This is because the autophagy inhibitor suppressed autophagy and prevented the AGEs-induced expression of β-catenin. This results in downregulation of the activity of the calcification pathway Wnt/β-catenin, resulting in the inhibition of cell calcification (Huang et al., 2016). This study found that in comparison to HASMCs exposed only to AGEs, HASMCs treated with an autophagy inhibitor significantly decreased pro-apoptotic protein's expression. This suggested that the suppression of apoptosis also accompanied the inhibition of autophagy, but the detailed mechanism needs further investigation. Our results confirmed that inhibition of autophagy is an effective way to prevent AGEs-induced calcification and apoptosis in HASMCs.

However, in some special circumstances, autophagy induction would facilitate apoptosis or necrosis stimulation (Redza-Dutordoir and Averill-Bates, 2016). In this study, with the dosage of AGEs increase, the autophagy markers, Beclin1 and LC3-II, were increased in HASMCs. The expression of p-mTOR and p62 decreased simultaneously. These results were suggesting that the AGEs treatment induced autophagy and calcification in HASMCs was consistent. We found that AGEs-induced autophagy, calcification, and apoptosis in HASMCs can be suppressed by treatment with an autophagy inhibitor or knockout of the autophagy-related gene Atg7, indicating that AGEs-induced autophagy triggers apoptosis. However, the detailed mechanism needed further investigation. The autophagy-relevant proteins may have additional roles in pro-apoptotic signaling. Atg7 is a protein upstream of LC3 in the mTOR pathway that can regulate autophagy and regulate apoptosis. The deletion of Atg5, Atg7, or Beclin1 can prevent cell death and restore cell clonality (Elgendy et al., 2011). In this study, the autophagy-related gene Atg7 was knocked out in HASMCs, followed by AGEs exposure. We found that compared with those in wild-type HASMCs, the expression levels of LC3-II and Atg7 proteins in Atg7 knockout HASMCs were significantly decreased or even absent. It was confirmed that autophagy in HASMCs was inhibited after Atg7 gene knockout, similar to autophagy inhibitor WM. Within the same concentration range of AGEs, the expression of proteins related to calcification and apoptosis in HASMCs with Atg7 knockout was significantly suppressed. This reflects that AGEs-induced autophagy of HASMCS could inhibit the knockout of the Atg7 gene, thus preventing calcification and apoptosis and protecting cells. The autophagy-relevant proteins may have additional roles in pro-apoptotic signaling. For instance, inhibit the Beclin 1 protein, which has crosstalk with the anti-apoptotic BCL-2 family proteins, could suppress the pro-autophagic process. However, this does not inflect with the anti-apoptotic progress of the BCL-2 family proteins. JUN N-terminal kinase can phosphorylate BCL-2, disrupting the inhibitory crosstalk with Beclin 1, leading to autophagy. Besides, this prevents BCL-2 from inhibiting pro-apoptotic proteins, thus promoting apoptosis. Nevertheless, further study is needed to investigate the key signaling molecule to lead to autophagy and apoptosis by AGEs in HASMCs.

**FIGURE 9:**
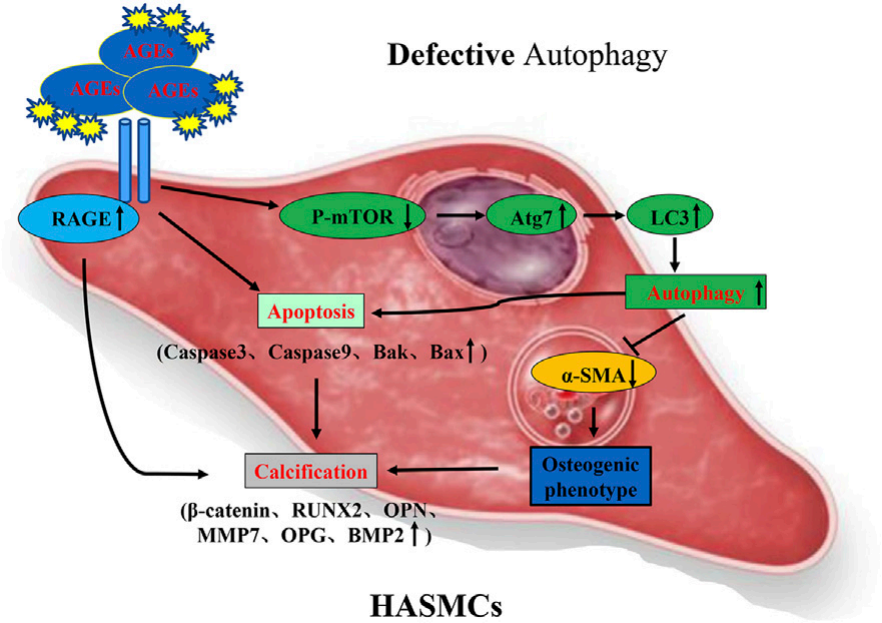
Possible mechanism of AGEs-induced calcification in HASMCs via the induction of autophagy and apoptosis.

Autophagy was also found to be involved in the process of phenotypic switching of HASMCs. By determining α-SMA levels (the smooth muscle cell-specific protein), we found that AGEs could reduce α-SMA expression. When cells were exposed to both WM and AGEs, the suppression of α-SMA was alleviated. We found that phenotypic transformation of SMC could be prevented by repressing autophagy via electron microscopic analysis (autophagosome detection) in the HASMCs. In summary, the autophagy inhibitor WM could be used to suppress autophagy induced by AGEs to prevent phenotypic switching (Figure 9).

The study has several limitations. 1) In this autophagy study, we did not use other autophagy inducers or inhibitors, which could stimulate HASMCs and bafilomycin A1 to detect the increase in autophagy flux. 2) In this study, we have explored the relationship between apoptosis and autophagy but did not explore the specific mutual regulation mechanism. 3) In this study, we could not perform the further calculation of the calcification area, all the fluorescence images could not be quantified, and the p62 detection was not presented. 4) In addition, cleaved-caspase3/caspse3 and cleaved-caspase9/caspase9 could not be tested. Therefore, we intend to conduct further experiments with an extended experimental plan in the future.

In conclusion, AGEs can induce calcification, autophagy, and apoptosis in HASMCs. AGEs-induced autophagy, calcification, and apoptosis in HASMCs can be suppressed by treatment with an autophagy inhibitor or knockout of the autophagy-related gene Atg7. Therefore, our results indicated that inhibition of AGEs-induced autophagy could prevent the apoptosis of HASMCs and the transformation of osteoblasts. Besides, autophagic cell death refers to cell death by autophagy. The final cell death process is mediated by autophagy rather than other cell death modalities, such as apoptosis or necroptosis. Thus, whether the autophagy in this paper is autophagic cell death needs further investigation. These results gave us new ideas to conduct further investigations to understand better whether there is any possibility of developing a new therapeutic strategy for the treatment of diabetes mellitus-associated vascular calcification.

## Data Availability

The raw data supporting the conclusions of this article will be made available by the authors, without undue reservation, to any qualified researcher.

## Abbreviations

AGEs, advanced glycation end products; ALP, alkaline phosphatase; Atg7, autophagy-related gene 7; DM, diabetes mellitus; HASMCs, human aortic smooth muscle cells; RAGE, AGEs receptors; SMA, the smooth muscle cell-specific protein; SMC, smooth muscle cells; T2DM, type 2 diabetes mellitus; VC, vascular calcification; VSMCs, vascular smooth muscle cells; WM, wortmannin
